# Contemporary Tailored Oncology Treatment of Biliary Tract Cancers

**DOI:** 10.1155/2019/7698786

**Published:** 2019-12-18

**Authors:** Fiona Turkes, Juliet Carmichael, David Cunningham, Naureen Starling

**Affiliations:** Department of Medicine, Royal Marsden Hospital NHS Foundation Trust, London, UK

## Abstract

Biliary tract cancers (BTCs) are poor prognosis malignancies with limited treatment options. Capecitabine has recently emerged as an effective agent in the adjuvant setting; however, treatment of advanced disease is still limited to first-line cisplatin and gemcitabine chemotherapy. Recent global efforts in genomic profiling and molecular subtyping of BTCs have uncovered a wealth of genomic aberrations which may carry prognostic significance and/or predict response to treatment, and several targeted agents have shown promising results in clinical trials. As such, the uptake of comprehensive genomic profiling for patients with BTCs and the expansion of basket trials to include these patients are growing. This review describes the currently approved systemic therapies for BTCs and provides insight into the emerging targeted and immunotherapeutic agents, as well as conventional chemotherapeutic regimes, currently being investigated in clinical trials.

## 1. Introduction

Biliary tract cancers (BTCs), encompassing cholangiocarcinoma (CCA) and gallbladder cancer (GBC), are relatively rare cancers with wide geographical diversity [1]. Cholangiocarcinomas are generally divided into intrahepatic (ICC) and extrahepatic tumours (ECC) with the latter further was separated into perihilar cholangiocarcinoma (PCC) and distal cholangiocarcinoma (DCC) [2]. The highest rates of CCA are seen in countries such as China and Thailand where liver fluke infection is prevalent [3, 4]. However, the incidence of CCA in most Western countries is also slowly rising [5], probably as a consequence of improved imaging techniques and diagnosis in addition to the increasing burden of chronic diseases such as fatty liver disease and viral hepatitis [6, 7]. On the other hand, the highest incidence of GBC is in Chile where gallstone disease is the primary risk factor [8].

Unfortunately, BTCs carry an extremely poor prognosis with an overall 5-year survival in the region of 5-15% [9]. The majority of patients present with unresectable or advanced disease at diagnosis [10]; thus, systemic therapy is their only treatment option. In addition to the diverse aetiological origins of BTCs, it is well recognised that the BTC subtypes also differ in their tumour biology [11–13] and clinical presentation [2]. For example, patients with ECC are more likely to present with obstructive jaundice, and thus, their disease may be diagnosed at an earlier stage. For those who do present with resectable disease, surgery may be curative; however, these patients represent a small minority and relapse rates are high [14]. Furthermore, the patterns of relapse usually preclude further curative resection [9, 15]. There is, therefore, a huge unmet need for more effective therapies for the treatment of BTCs.

This review describes the currently approved systemic therapies for BTCs and, following recent advances in the molecular profiling of these rare tumours, provides insight into some of the promising new agents under investigation in clinical trials, with the goal of improving patient outcomes.

## 2. Systemic Adjuvant Treatment for Resected Biliary Tract Cancers

The first randomised controlled trial to assess the benefit of adjuvant chemotherapy in resected BTCs was run in Japan and included patients with resected carcinoma of the pancreas and ampulla of Vater [16]. The group found that the 5-year survival rate of patients with gallbladder cancer who received postoperative mitomycin C and 5-flurouracil (*n* = 69) was significantly better compared to that of patients who received surgery alone (*n* = 43) in the per-protocol analysis but not the intention-to-treat analysis (26.6% vs. 14.4%) (*p* = 0.0367). However, no significant benefit was seen in any other tumour types and the improved survival of patients with gallbladder cancer who received chemotherapy was confined to those who had “noncurative” resections [16]. A subsequent meta-analysis of data from ten nonrandomised retrospective studies, including a total of 3191 patients with gallbladder cancer, supported a survival benefit for postoperative chemotherapy overall (HR = 0.42); however, the benefit of all adjuvant treatments (including radiotherapy and chemoradiotherapy) was limited to patients with node- and margin-positive diseases only [17]. Furthermore, the survival benefit of adjuvant treatment appeared to be restricted to patients from Asia [17]. A second meta-analysis, including all BTC subgroups, only identified a survival advantage for adjuvant chemotherapy in patients with resected node-positive disease; however, the trials included were also mostly nonrandomised, retrospective studies [18]. The lack of available robust and prospective data supporting the use of adjuvant chemotherapy in resected BTCs meant that worldwide practice varied.

The multicentre phase III PRODIGE-12 study, which randomly allocated 196 patients with resected BTCs to either combination gemcitabine and oxaliplatin (GEMOX) chemotherapy or surveillance, reported in 2017 [19]. Patient stratification factors included tumour location split into ICC, ECC, or GBC; R0 versus R1 resection; and lymph node involvement or not. The trial was negative for the coprimary endpoint of relapse-free survival (RFS) with a median of 20.4 months in the GEMOX arm compared to 18.5 months in the surveillance arm (HR 0.88; 95% CI 0.62-1.25; *p* = 0.48). The authors attribute the likely reason for this lack of difference in RFS to an ambitious hazard ratio which was set at 0.6. Furthermore, in the preplanned subgroup analysis looking specifically at outcomes in the GBC cohort, there were significantly worse RFS (HR 2.56) and median overall survival (OS) (HR 3.39) in patients with GBC who received GEMOX (*n* = 17) compared to surveillance (*n* = 21).

The BILCAP study also reported in 2017. In this larger phase III U.K. study, 447 patients with resected BTCs were randomised to either capecitabine for 24 weeks or observation [20]. Most commonly, patients with DCC followed by ECC or PCC were recruited; in contrast, only 8% of the patients in the PRODIGE-12 study had PCC. BILCAP did not reach statistical significance for the primary endpoint of OS in the intention-to-treat population; however, a significant OS difference was observed in the per-protocol population (only 17 patients less than the intention-to-treat population) with a median OS of 53 months in the capecitabine arm compared to 36 months in the observation arm (HR 0.75). The RFS in the per-protocol analysis was also significantly longer in the interventional group compared to surveillance (HR 0.71). Of interest, in preplanned subgroup analyses, there was a significant benefit of capecitabine in men and those with poorly differentiated disease. There was also a trend towards benefit in lymph node-positive patients; however, this was just outside the level of statistical significance. Treatment was well-tolerated, and there were no significant differences in the quality of life between the two study arms. The updated ASCO guidelines now recommend 6 months of adjuvant capecitabine for all patients with resected biliary tract cancer [21] whereas the NCCN continues to advocate for individualised decisions and recommend varying strategies dependent upon the BTC subtype, lymph node involvement, and resection margin status [22].

The authors of the BILCAP study highlight that because BTC is a rare disease, and of course resectable BTC even more so, the study needed 10 years to fully accrue. PRODIGE-12 required fewer patients, but recruitment still took 5 years. In the meantime, it has become clearer that not all biliary tumours are born equal, and while males or those with poorly differentiated tumours or lymph node involvement may benefit the most from adjuvant chemotherapy, other patients such as those with GBC who had the worst outcomes in PRODIGE-12 may require a different treatment strategy altogether. Additionally, it is also important to bear in mind that PRODIGE-12 and BILCAP only recruited from centres in Europe whereas some of the highest incidences of BTCs are seen in Asia. Given the diverse aetiologies of these tumours, we should be mindful of the applicability of BILCAP worldwide. The BCAT study was another phase III study which assessed the benefit of single-agent gemcitabine over observation in patients with resected BTCs recruited from 48 Japanese centres [23]. BCAT restricted inclusion to patients with PCC or DCC. Again, adjuvant gemcitabine failed to show a significant advantage in improving OS compared to placebo [23].  Table 1 summarises the completed positive and negative trials investigating adjuvant therapies following resection of BTCs.

The ongoing randomised ACTICCA-1 study recently changed its control arm from surveillance to capecitabine following the results of BILCAP. It will determine whether there is added clinical benefit of intensification of chemotherapy with cisplatin plus gemcitabine in the adjuvant setting compared to single-agent capecitabine. Given the results of PRODIGE-12, BILCAP, and BCAT thus far, it seems that future study design should also concentrate on determining which specific characteristics, global populations, or BTC subtypes would most meaningfully benefit from adjuvant chemotherapy and its intensification or not.  Table 2 summarises some ongoing clinical trials investigating adjuvant therapies following resection of BTCs.

## 3. Systemic Treatment of Advanced Disease

### 3.1. Chemotherapy

#### 3.1.1. First-Line Chemotherapy

The ABC-02 study, conducted across 37 U.K. centres, established cisplatin and gemcitabine as the standard of care regimen for the first-line treatment of advanced BTCs in 2010 [24]. In this randomised phase III study, 410 patients with advanced BTCs were allocated to receive either single-agent gemcitabine or the cisplatin/gemcitabine doublet and there was a confirmed significant OS advantage of cisplatin/gemcitabine over the single agent (11.7 vs. 8.1 months; HR 0.64) (95% CI 0.52–0.80; *p* < 0.001) [24]. A corresponding randomised study in Japanese patients confirmed benefit in this population [25]. However, a subsequent meta-analysis of these two studies suggested that patients with poor performance status may not derive benefit from the doublet [26], and so, current guidelines permit the use of single-agent gemcitabine in these patients [2]. Cisplatin may also be substituted by oxaliplatin in cases of renal impairment [2].

Since the pivotal results of ABC-02, a number of phase II and III studies have assessed the potentially added benefit of combining various targeted agents, e.g., erlotinib [27], panitumumab [28–30], cetuximab [31, 32], bevacizumab [33], and cediranib [34], with doublet chemotherapy. However, in most cases, these were unselected populations and results were either negative or not convincing enough to alter clinical practice. A retrospective analysis of 42 patients with advanced BTCs treated with FOLFIRINOX chemotherapy in the first line suggested that the triplet regimen was efficacious without increased toxicity [35]. The prospective phase II/III AMEBICA study will investigate whether intensification of chemotherapy with FOLFIRINOX will further improve outcomes compared to gemcitabine/cisplatin in the first line advanced setting [36] (Table 3).

#### 3.1.2. Second-Line Chemotherapy

Due to the aggressive nature of advanced BTC and problems with recurrent biliary obstruction; it has been historically difficult to robustly assess further treatment in trials after progression on cisplatin/gemcitabine. There had been some evidence to suggest possible benefit from second-line 5-FU chemotherapy in fit patients [37, 38], but the results of the U.K.-led phase III randomised ABC-06 study have since confirmed an overall survival benefit of FOLFOX (5-FU and oxaliplatin) chemotherapy plus best supportive care (BSC) compared to BSC alone [39] (6.2 vs. 5.3 months; HR 0.69) (95% CI 0.50–0.97; *p* = 0.031). Following these results, FOLFOX plus BSC may well become the established second-line regimen for fit patients with advanced BTC.

### 3.2. Genomic Profiling of Biliary Tract Cancers

Recent genomic sequencing data from across the world have shown that BTCs display a diverse mutational landscape [11–13]. Almost half of patients with BTCs have been shown to harbour at least one driver mutation which may represent a therapeutic opportunity and/or a prognostic biomarker [13]. Given that studies of targeted agents in unselected populations have not shown significant benefit, these genomic data represent a novel approach for trials of targeted therapies in biomarker-enriched populations.

Javle et al. were the first group to correlate genomic mutational patterns, using the FoundationOne platform, with clinical outcomes [11]. They found that the most commonly aberrant genes varied depending on BTC subtype—TP53 (27%) in ICC, KRAS (42%) in ECC, and ERBB2 (16%) in GBC, and that FGFR mutations, mostly detected in ICC, were associated with a good prognosis [11]. Subsequently, by performing integrative clustering analysis of mutation, copy number, gene expression, and epigenetic data on tissue from nearly 500 CCAs, four different and distinct molecular subtypes of CCA have emerged [12]. Cluster 1 are mostly fluke-positive tumours enriched with TP53 and ARID1A gene alterations, ERBB2 amplification, and CpG island hypermethylation, whereas Cluster 4 are mostly fluke-negative ICC enriched with FGFR alterations and CpG shore hypermethylation [12]. Interestingly, this group also showed that the molecular rather than anatomic subtype of CCA has much more of a bearing on prognosis, with Cluster 4 tumours in the better prognosis category [12]. This is in keeping with findings from Javle et al. who showed that FGFR mutations were associated with improved OS [11]. A prospective analysis using the MSK-IMPACT platform also identified distinct molecular patterns between ICC and ECC [13].

These genetic alterations and distinct molecular subtypes present potential therapeutic targets which could be exploited by targeted agents and support the implementation of a platform for genomic profiling to be available to all patients with advanced BTCs. The MOSCATO-1 trial was a large-scale prospective study which performed genomic analyses on over 1000 tumour samples and matched 199 patients to a targeted therapy based on a genetic alteration, 18 of whom had advanced BTC and had been treated with at least one prior line of systemic treatment [40]. In these 18 who received a matched targeted therapy, the overall response rate was 33% and progression-free survival (PFS) was 5.2 months [41]. Furthermore, the median OS of those who received a targeted treatment was 17 months, which far surpasses the median OS in the second-line setting so far reported [37]. A much higher rate of potentially actionable mutations was also seen in BTCs compared to other tumour types analysed in the MOSCATO-1 study [41].

Given that sequencing of tissue samples can be limited by low tumour content, liquid biopsy is also being harnessed for genomic profiling in BTC. Circulating tumour DNA (ctDNA) analysis using the Guardant 360 assay on 138 patient samples detected at least 1 genomic alteration in 89% of patients—most commonly, TP53, KRAS, and FGFR2 [42], although concordance with tissue-based alterations in BTC has yet to be proven. A nationwide ctDNA genomic screening platform using the Guardant 360 assay is currently recruiting in Japan and enrolling patients into clinical trials based on targetable genomic alterations [43].

### 3.3. Emerging Therapeutic Targets

#### 3.3.1. FGFR Fusions

FGFR gene fusions are present in approximately 15% of patients with mostly noninfectious ICC [44], the most common fusion partners being BICC1 and KIAA1217 [13]. They result in the activation of canonical downstream signalling and have been associated with improved survival [11]. Infigratinib (BGJ398) is a potent oral FGFR1-3 kinase inhibitor which was first tested in patients with CCA and FGFR aberrations in a phase II study [45]. Sixty-one patients with advanced CCA and FGFR aberrations (mostly FGFR fusions, *n* = 48) were treated with the agent in the second- or later-line setting, and results demonstrated an ORR of 14.8% and DCR of 75.4%. Interestingly, all patients who experienced a radiological response had an FGFR2 fusion suggesting that BGJ398 was particularly sensitive to this oncogenic driver. Furthermore, the side effect profile including hyperphosphataemia, fatigue, and stomatitis was manageable. Preliminary results of a phase II study of erdafitinib, another potent oral pan-FGFR tyrosine kinase inhibitor, tested in Asian patients with CCAs and FGFR alterations also indicate an ORR (CR and PR) of 45.5% in 11 evaluable patients which is encouraging [46]. Side effects from erdafitinib were similar to those from BGJ398, most commonly, hyperphosphataemia, stomatitis, dry skin, and nail disorders [45, 46]. Hyperphosphataemia appears to be a class effect on FGF23 which is involved in phosphate metabolism [47]. INCB054828, another pan-FGFR, is currently being investigated in a phase III trial against gemcitabine and cisplatin after demonstrating promising early activity in patients with CCA (Table 3). ARQ087 (derazantinib), a nonselective multikinase inhibitor which includes the FGFR as a target, has also recently entered phase III testing in pretreated patients after phase II data from 29 patients with ICC, and FGFR fusions treated with derazantinib revealed a median PFS of 5.7 months (95% CI: 4.04–9.2 months) and an ORR of 20.7% [48].

As frequently seen with kinase inhibitors, however, secondary acquired resistance eventually ensues. For the BGJ398 drug, the mechanism of acquired resistance has been identified as the development of a polyclonal point mutation in the FGFR2 kinase domain from serial analysis of tissue and cell-free circulating DNA (cfDNA) from patients on treatment and at disease progression [49]. Another postulated escape mechanism included the PI3K/PTEN pathway [49]. TAS-120 is a highly selective covalent pan-FGFR inhibitor which is active against FGFR2 resistance mutations. Following promising clinical activity in FGFR aberrant ICC in an early-phase study [50], the phase II FOENIX-101 study of TAS-120 in patients with ICC harbouring FGFR2 gene rearrangements after progression on first-line treatment is currently recruiting (Table 3). Going forward, there may also be a rationale to combine FGFR2 inhibition with agents that target the PI3K/PTEN pathway.

#### 3.3.2. IDH Mutations

IDH1 and IDH2 mutations are present in approximately 20% of mainly noninfectious ICC [12, 13, 51, 52]. They result in the accumulation of 2-hydroxyglutarate (2-HG) which drives tumourigenesis and can be measured in tumour and blood [53]. Ivosidenib (AG-120) is a first in class oral, selective, reversible IDH1 inhibitor which first showed efficacy in patients with advanced IDH-1-mutated CCA (*n* = 73) in a phase I study of solid tumours with IDH1 mutations [54]. 6% of patients had a partial response to treatment, and the other 56% had stable disease. There were no dose-limiting toxicities, and the main side effects, including fatigue and nausea, were manageable. The randomised phase III placebo-controlled ClarIDHy trial results have since demonstrated a PFS advantage in patients with IDH1-mutated CCA who have failed prior treatment who took ivosidenib 500 mg four times a day over placebo; 2.7 months compared to 1.4 months (HR 0.37; 95% CI 0.25–0.54; *p* < 0.001) [55]. The disease control rate (stable disease plus partial response) was also superior in the ivosidenib arm at 53% compared to the placebo arm at 28%; there was a trend towards OS benefit, and the main side effects associated with ivosidenib including mostly gastrointestinal toxicities such as nausea and diarrhoea and fatigue were manageable. The ClarIDHy trial results are the first phase III data to show benefit from IDH1 inhibition in patients with IDH1-mutated CCA. IDH1-mutated ICC cells have been shown to be exquisitely sensitive to dasatinib [56], a multitargeted TKI currently approved to treat certain leukaemias, and results of a phase II trial in patients with IDH-mutant advanced ICC are awaited (Table 3). BAY1436032 is another recently developed drug which is being investigated in an IDH1 mutation basket study with a CCA cohort (Table 3). Given that 2-HG can hamper homologous recombination and has demonstrated sensitivity to PARP inhibition in preclinical models [57], another IDH1 mutation basket study with a CCA cohort is also assessing whether the PARP inhibitor olaparib can affect ORR (Table 3).

#### 3.3.3. DNA Damage Repair Mechanisms and BAP1 Mutations

The DNA damage repair (DDR) pathways are essential for maintaining genomic integrity by promoting DNA repair, cell cycle arrest, and apoptosis. Somatic or germline alterations to the DDR genes not only have been linked to carcinogenesis but also represent increased sensitivity to DNA-damaging agents which can be exploited in cancer therapy. The prevalence of mutations in DDR genes is generally low in BTCs; however, mutations in BAP1, which is a tumour suppressor gene involved in DNA double-strand break repair associated with noninfectious CCA [12], have emerged as a potential target [58]. A phase II basket trial is currently investigating the clinical benefit of the PARP inhibitor (niraparib) in patients with BAP1 mutations and other DDR-deficient cancers including CCA (Table 3).

#### 3.3.4. Mismatch Repair Deficiency, Microsatellite Instability, and Tumour Mutational Burden

It is widely recognised that tumours which exhibit deficient mismatch repair (dMMR) expression and an unstable microsatellite (MSI) phenotype, via either germline or somatic mutations, have favourable responses to checkpoint inhibiting immunotherapy. As such, the PD1 inhibitor pembrolizumab was given the first tumour-agnostic approval by the FDA in 2017 for the treatment of any dMMR or MSI-high tumour [59]. While the rate of dMMR/MSI in CCA is only in the region of 2.5% [60], upregulated immune-related pathways including PD1 have been reported in one of the molecular subtypes of BTC (Cluster 3) [12] which may also confer benefit from treatment with anti-PD1 therapy. Indeed, in the KEYNOTE-028 basket study of pembrolizumab in biomarker-selected patients, 17% of patients with PDL1-positive tumours in the BTC cohort achieved a partial response [61]. KEYNOTE-016 and KEYNOTE-158 also assessed the benefit of pembrolizumab in dMMR/MSI-high tumours and achieved an ORR of 53% and 37% in the BTC cohorts, respectively [60, 62]; responses were also durable with a 2-year OS of 64% (95% CI: 53–79) in KEYNOTE-016 [60]. In contrast, the ORR for unselected BTC patients in KEYNOTE-158 (*n* = 104) was only 5.8% [63]. These results support testing for dMMR/MSI in all patients with advanced BTC.

High tumour mutational burden (TMB) has also been shown to predict response to immune checkpoint inhibition due to increased neoantigen presentation [64]. In a retrospective analysis of patients with solid tumours (mostly lung and melanoma), 42% of those with high TMB, defined as over >20 mutations/megabase (Mb), had an objective response to checkpoint inhibiting therapy compared to only 2/46 with low TMB [65]. CHECKMATE-848 is a currently recruiting randomised study of combination checkpoint inhibition with nivolumab, anti-PD1, and ipilimumab, anti-CTLA4, or nivolumab alone in patients with solid tumours, including BTC, with high TMB defined as >15 mutations/Mb (Table 3). It will determine whether the combination strategy leads to increased clinical benefit as seen in other immunogenically “hot” malignancies such as melanoma and, for patient benefit, crossover is allowed. Given the promising responses to immunotherapy thus far, there is, of course, a strong rationale for combining checkpoint inhibition with chemotherapy and the randomised phase III TOPAZ-1 study will assess whether gemcitabine and capecitabine with durvalumab, anti-PDL1, will improve OS compared to placebo in 1^st^ line unselected patients with BTC (Table 3).

The role of adoptive immunotherapy, whereby a patient's own tumour-infiltrating lymphocytes are harvested and then infused back into them to boost the immune response, is also currently being investigated in a number of clinical trials in patients with biliary tract cancers (Table 3). In one case report, a patient with lymph node-positive disease and portal vein invasion at surgery was treated with CD3-activated T cells and dendritic cells in the adjuvant setting and was still alive more than 3 and a half years later [66]. Another patient with a heavy burden of metastatic disease was treated with CD4+ T cells which recognised an erbb2 epitope on the cancer cell and experienced a durable response to treatment which was recapitulated on disease progression [67].

### 3.4. Other Potential Targets

#### 3.4.1. ERBB2 (HER2) Aberrations

ERBB2/HER2 aberrations have been detected in 3.9-8.5% of most commonly fluke-positive tumours CCAs and 16% of GBC and have been associated with poorer prognosis [11, 12]. There are several HER2-directed agents with well-defined safety profiles already approved to treat a number of malignancies such as breast and gastric cancer; however, thus far, the only indication of a signal in patients with BTCs harbouring HER2 aberrations treated with HER2-directed therapy has been in retrospective series [68], and efficacy is yet to be confirmed in prospective trials. The HERB trial is a currently recruiting phase II trial of the HER2 inhibitor, DS-8201a, in patients with HER2-positive (1+ by IHC and positive by ISH) biliary tract cancer in Japan (Table 3).

#### 3.4.2. RAS/MAPK Pathway

KRAS is a key oncogenic driver in many malignancies and has been proven notoriously difficult to target due to the number of different proteins it interacts with both directly and indirectly. Most developed agents therefore target downstream proteins in the signalling pathway such as BRAF or MEK. MEK inhibitors have had limited activity as single agents in BTCs [69]. There has however been efficacy reported with the MEK inhibitor selumetinib in combination with cisplatin/gemcitabine chemotherapy in a phase Ib study, and the side effects were tolerable [70]. There are also a number of BRAF and MEK inhibitor combination studies currently ongoing for patients with BRAF-V600E-mutated advanced solid tumours (Table 3). The preliminary results of the biliary tract cohort with BRAF-V600E mutations from the ROAR trial show encouraging efficacy with dabrafenib (BRAF inhibitor) and trametinib (MEK inhibitor). In 32 evaluable patients in the BTC cohort, the ORR was 41% and the median OS reached 11.3 months (95% CI, 7.3–17.6) [71]. As MEK inhibitors have been shown to increase immune recognition of tumour cells and promote T cell survival and accumulation [72], there is also rationale to combine them with immunotherapeutic agents; however, a recent phase III trial assessing the combination of cobimetinib (MEK inhibitor) and atezolizumab (PDL1-inhibitor) against the standard of care in patients with microsatellite stable (MSS) colorectal cancer did not improve OS [73].

#### 3.4.3. PI3K/AKT/mTOR

Aberrations in the PI3K/AKT/mTOR pathway, such as PI3K mutations, PI3KCA amplifications, phosphorylated AKT (p-AKT), and p-mTOR overexpression, have been detected in BTCs and are associated with poorer prognosis [74]. The loss of expression of PTEN, a tumour suppressor gene involved in the regulation of the PI3K/AKT/mTOR pathway, has also been found in 4.1-51.8% of GBC [75, 76]. Thus far, early-phase clinical studies of an AKT inhibitor (MK-2206) [77], an mTOR inhibitor (everolimus) [78], and a PI3K inhibitor (buparlisib) together with FOLFOX [79] have shown limited tumour responses. Postulated reasons for these disappointing results include the lack of robust molecular stratification in these initial studies, likely resistance mechanisms related to the use of single targeted agents, and the small patient population suitable for clinical trial entry [74].

#### 3.4.4. NTRK Fusions

The FDA has recently granted a second tumour-agnostic approval to larotrectinib, a neurotrophic receptor tyrosine kinase (NTRK) inhibitor, for patients with solid malignancies and a proven NTRK gene fusion without a known acquired resistance mutation [80]. The accelerated approval was based on efficacy data from 55 patients from the NAVIGATE trial with 12 different pretreated solid malignancies harbouring NTRK fusions, 22% of whom demonstrated a complete response and 53% a partial response to treatment with 73% of patients experiencing a maintained response for more than 6 months [81]. However, while NTRK fusions have been characterised in patients with ICC in Asia [82], NTRK fusions were not identified in a pooled cohort of 106 Caucasian patients [83]. The NAVIGATE trial is currently still recruiting as is a basket study assessing the benefit of entrectinib, another NTRK inhibitor, in patients with advanced solid tumours harbouring NTRK1/2/3 or ROS1 or ALK gene fusions (Table 3).

## 4. Conclusion

Recently, a role for empirical capecitabine chemotherapy in the adjuvant setting has been defined. However, in the advanced setting, there has been a concerted move towards adoption of a truly personalised approach to treatment by selecting appropriate targeted therapies based on particular molecular aberrations specific to an individual patient's tumour. This has been possible though global advances in genomic profiling and molecular subtyping of BTCs which have broadened our understanding of their hugely complex molecular landscape and the potential “druggable” targets which could be exploited. The evolution of histology-independent basket trials, where patients can be enrolled into studies based on a specific molecular aberration rather than tumour type, has also been vital in order to assess the potential benefit of these targeted therapies in rare cancers such as BTCs. So far, therapies targeting FGFR2 fusions and IDH mutations have gone the farthest in trials with the most promising results; however, a deeper understanding of potential resistance mechanisms and the complex crosstalk between molecular pathways is growing and combination strategies targeting more than one pathway are being proposed. In order to benefit from tailored therapy, genomic testing for all patients with BTC should be considered and liquid biopsy may be the most convenient way to implement this.

## Figures and Tables

**Table 1:** Completed clinical trials investigating adjuvant therapies following resection of biliary tract cancers.

Trial	Study arms	Phase	Tumour site	Resection margins	Nodal status	Overall survival (months)	Disease recurrence
Positive trials							
BILCAP [20]	Capecitabine × 8 vs. observation (1 : 1)	III Open-label *n* = 447	ICC: *n* = 84 (19%) PCC: *n* = 128 (29%) DCC: *n* = 156 (35%) GBC: *n* = 79 (18%)	R0: *n* = 279 (62%) R1: *n* = 168 (38%)	N0: *n* = 236 (53%) N1: *n* = 210 (47%)	*ITT:* 51·1 vs. 36·4 HR 0·81 [95% CI 0·63–1·04]; *p* = 0 · 097 *PPA:* 53 vs. 36 HR 0·75 [95% CI 0·58–0·97]; *p* = 0 · 028	*Median RFS (months)* *ITT:* 24·4 vs. 17·5 HR 0.75 [95% CI 0·58–0·98]; *p* = 0 · 033 *PPA*: 25.9 vs. 17.4 HR 0·70 [95% CI 0·54–0·9]; *p* = 0 · 0093

Negative trials							
Takada et al. [16]	Mitomycin C and 5-flurouracil vs. surgery alone	III Open-label *n* = 508	Pancreas: *n* = 173 (34%) Bile duct: *n* = 139 (27%) GBC: *n* = 140 (28%) Ampulla of Vater: *n* = 56 (11%)	Curative: *n* = 256 Noncurative: *n* = 180	Not reported	*ITT (GBC cohort only):* 16.4 vs. 14.1 (*p* = 0.28) *PPA of 5-year survival rate (%) in GBC cohort:* 26.0% vs. 14.4%, (*p* = 0.0367^∗^)	*5-year DFS rate (%) in GBC cohort:* 20.3% vs. 11.6%; *p* = 0.0254^∗^
PRODIGE 12-ACCORD 18 [19]	GEMOX × 12 vs. observation	III Open-label *n* = 194	ICC: *n* = 86 (44%) PCC: *n* = 15 (8%) DCC: *n* = 55 (28%) GBC: *n* = 38 (20%)	R0: *n* = 169 (87%) R1: *n* = 25 (13%)	N0: *n* = 97 (50%) N1: *n* = 69 (36%) N2: *n* = 2 (1%) Nx: *n* = 26 (13%)	75.8 vs. 50.8 HR 1.08 [95% CI 0.70-1.66]; *p* = 0.074	*Median RFS (months):* 30.4 vs. 18.5 HR 0.88 [95% CI 0.62-1.25]; *p* = 0.48
BCAT [23]	Gemcitabine × 6 vs. observation (1 : 1)	III Open-label *n* = 225	PCC: *n* = 102 (45%) DCC: *n* = 123 (55%)	R0: *n* = 204 (91%) R1: *n* = 25 (11%)	N0: *n* = 147 (65%) N1: *n* = 78 (35%)	62.3 vs. 63.8 HR 1.01 [95% CI 0.70–1.45]; *p* = 0.964	*Median RFS (months):* 36.0 vs. 36.9 HR 0.93 [95% CI 0.66–1.32]; *p* = 0.693
ESPAC-3 [84]	Fluorouracil/folinic acid vs. gemcitabine vs. observation (1 : 1 : 1)	III Open-label *n* = 428	Ampullary: *n* = 297 (69%) Bile duct: *n* = 96 (22%) Other: *n* = 35 (8%)	R0: *n* = 360 (84%) R1: *n* = 68 (16%)	N0: *n* = 177 (41%) N1: *n* = 251 (59%)	38.9 vs. 45.7 vs. 35.2 HR for FU vs. observation: 0.95 [95% CI 0.71-1.28]; *p* = 0.74 HR for gemcitabine vs. observation: 0.77 [95% CI 0.57-1.05]; *p* = 0.10	*Median RFS (months):* 23.0 vs. 29.1 vs. 19.5 HR for FU vs. observation: 0.69 [95% CI 0.51-0.95]; *p* = 0.02 HR for gemcitabine vs. observation: 0.68 [95% CI 0.50-0.95]; *p* = 0.02

OS: overall survival; RFS: relapse-free survival; ICC: intrahepatic cholangiocarcinoma; PCC: perihilar cholangiocarcinoma; DCC: distal cholangiocarcinoma; GBC: gallbladder carcinoma; ITT: intention-to-treat; PPA: per protocol analysis; HR: hazard ratio; 95% CI: 95% confidence interval; GEMOX: gemcitabine+oxaliplatin. ^∗^This subgroup analysis was statistically significant, and therefore, there is a positive finding in the study.

**Table 2:** On-going clinical trials investigating adjuvant therapies following resection of biliary tract cancers.

Agent [trial]	Mechanism of action	Population	Phase	Treatment arms	Planned recruitment	Primary endpoint	Clinical trial identifier
GEM/CIS [ACTICCA-1]	Cytotoxic	Resected localised biliary tract cancer, following complete macroscopic resection	III Open-label	GEM/CIS × 8 vs. capecitabine × 8 (1 : 1)	Recruiting Target *n* = 781	DFS	NCT02170090
GEM/CAP [AdBTC-1]	Cytotoxic	Resected localised biliary tract cancer, following complete macroscopic resection	III Open-label	GEMCAP × 8 vs. capecitabine × 8	Recruiting Target *n* = 460	DFS	NCT03779035
S-1 [JCOG1202, ASCOT]	Cytotoxic	Resected localised biliary tract cancer, following complete macroscopic resection	III Open-label	S1 × 4 vs. observation	Recruiting Target *n* = 440	OS	UMIN000011688
Apatinib	VEGFR2	Resected biliary tract cancer	II Open-label	Apatinib plus capecitabine vs. capecitabine	Not yet recruiting Target *n* = 40	PFS	NCT03609489
Nivolumab+cabrilizumab	PD1; CSF1	Confirmed biliary tract cancer	II Single-arm	Nivolumab+cabrilizumab	Not yet recruiting Target *n* = 16	Drug-related toxicity	NCT03768531

GEM/CIS: gemcitabine+cisplatin; GEMCAP: gemcitabine+capecitabine; DFS: disease-free survival; OS: overall survival; VEGFR2: vascular endothelial growth factor receptor 2; PFS: progression-free survival; PD1: programmed death 1; CSF1: colony-stimulating factor 1.

**Table 3:** On-going clinical trials investigating therapies for advanced biliary tract cancers.

Agent [trial]	Mechanism of action	Population	Phase	Treatment arms	Planned recruitment	Primary endpoint	Clinical trial identifier
Intensified cytotoxic therapies							
Nab-paclitaxel	Cytotoxic (antimicrotubule)	2^nd^ line Unresectable advanced or metastatic biliary tract and gall bladder carcinoma	III Open-label	GEM/CIS+nab-paclitaxel vs. GEM/CIS	Recruiting, target *n* = 268	OS	NCT03768414
mFOLFIRINOX [AMEBICA]	Combination chemotherapy	2^nd^ line Unresectable advanced or metastatic biliary tract and gall bladder carcinoma	II/III Open-label	mFOLFIRINOX vs. GEM/CIS	Recruiting, target *n* = 316	II: % alive at 6 months without radiological progression III: OS	NCT02591030

Agents targeting FGFR aberrations							
Ponatinib	Multitargeted TKI (including FGFR2)	Advanced biliary tract cancer harbouring FGFR2 fusion or amplification, 2^nd^ line or more	II Single-arm	Ponatinib	Active, not recruiting; *n* = 12	Clinical benefit rate	NCT02265341
Erdafitinib	Pan-FGFR TKI	Advanced solid tumours, including cholangiocarcinoma, with FGFR2 abnormalities	II Single-arm	Erdafitinib	Active, recruiting, target *n* = 55	ORR	NCT02699606
Derazantinib (ARQ087) [FIDES-01]	Multikinase inhibitor	Advanced/inoperable intrahepatic cholangiocarcinoma with FGFR2 fusion, 2^nd^ line or more	II Single-arm	Derazantinib	Active, recruiting, target *n* = 100	ORR	NCT03230318
TAS-120 [FOENIX101]	Pan-FGFR inhibitor	Advanced solid tumours, including intrahepatic/extrahepatic cholangiocarcinoma, with FGFR2 gene alteration, 2^nd^ line or more	II Single-arm	TAS-120	Recruiting, target *n* = 371	ORR	NCT02052778
Infigratinib (BJG 398) [PROOF]	FGFR1-3 kinase inhibitor	FGFR2 mutated (fusion/translocations) advanced cholangiocarcinoma, 1^st^ line	III Open-label	Infigratinib vs. GEM/CIS	Active, recruiting, target *n* = 350	PFS	NCT03773302
Pemigatinib (INCB054828) [FIGHT302]	Pan-FGFR TKI	FGFR2 rearranged advanced/irresectable cholangiocarcinoma	III Open-label	Pemigatinib vs. GEM/CIS	Active, recruiting, target *n* = 432	PFS	NCT03656536

Agents targeting IDH1/IDH2 mutations							
BAY1436032	IDH1 inhibitor	IDH-1 mutant advanced solid tumours	I	BAY1436032	Active, not recruiting, *n* = 81	MTD, no. of pts with AEs, established dose for phase II	NCT02746081
Dasatinib	Multitargeted TKI	IDH-1 mutant advanced intrahepatic cholangiocarcinoma	II	Dasatinib	Completed (results awaited), *n* = 8	ORR	NCT02428855
Olaparib	PARP inhibitor	IDH-1/IDH-2 mutant advanced solid tumours	II Single-arm	Olaparib	Recruiting, target *n* = 145	ORR	NCT03212274

Immunotherapy							
Pembrolizumab [ABC-09]	Anti-PD1	2^nd^ line Unresectable or metastatic biliary tract and gall bladder carcinoma	II Single-arm	GEM/CIS+pembrolizumab	Not yet recruiting, target *n* = 50	6-month PFS	NCT03260712
Durvalumab Tremelimumab	Anti-PD-L1 Anti-CTLA4	2^nd^ line Unresectable or metastatic biliary tract and gall bladder carcinoma	II Single-arm	Durvalumab+tremelimumab+GEM/CIS	Recruiting, target *n* = 31	ORR	NCT03046862
Nivolumab Ipilimumab	Anti-PD1 Anti-CTLA4	2^nd^ line Unresectable advanced or metastatic biliary tract and gall bladder carcinoma	II Open-label	Nivolumab/ipilimumab vs. GEM/CIS+nivolumab	Recruiting, target *n* = 64	PFS	NCT03101566
Nivolumab	Anti-PD1	2^nd^ line Unresectable or metastatic biliary tract and gall bladder carcinoma	II Single-arm	Nivolumab	Active, not recruiting, *n* = 52	ORR at 8 weeks	NCT02829918
Pembrolizumab	Anti-PD1	2^nd^ line or more, advanced irresectable biliary tract cancer	Phase II Single-arm	Pembrolizumab+CAPOX	Recruiting, target *n* = 19	5-month PFS	NCT03111732
Ipilimumab+nivolumab [CHECKMATE848]	Anti-PD1 Anti-CTLA4	Advanced or metastatic TMB-H solid tumours	Phase II Open-label	Ipilimumab+nivolumab vs. nivolumab	Recruiting, target *n* = 159	ORR	NCT03668119
Nivolumab Ipilimumab	Anti-PD1 Anti-CTLA4	Rare tumours (2^nd^ line or more)	II Single-arm	Nivolumab+ipilimumab	Recruiting, target *n* = 120	Clinical benefit rate	NCT02923934
Nivolumab Ipilimumab [DART]	Anti-PD1 Anti-CTLA4	Advanced GI tumours (2^nd^ line or more)	II Single-arm	Nivolumab+ipilimumab	Recruiting, target *n* = 707	ORR	NCT02834013
Durvalumab [TOPAZ-1]	Anti-PD-L1	2^nd^ line Unresectable or metastatic biliary tract and gall bladder carcinoma	III Double-blind	GEM/CIS+durvalumab vs. GEM/CIS+placebo	Recruiting, target *n* = 474	OS	NCT03875235
Autologous tumour-infiltrating lymphocytes+pembrolizmab	TIL Anti-PD1	Metastatic cancer including hepatobiliary (refractory to standard therapy)	II Multiarm	(i) CD enriched TIL (ii) Unselected TIL (iii) Unselected TIL+pembrolizumab prior to cells (iv) Unselected TIL+pembrolizumab at disease progression	Recruiting, target *n* = 332	ORR	NCT01174121
Modified autologous cytokine-induced killer cells	Cytokine-induced killer cells	Cholangiocarcinoma	I/II Single-arm	Cytokine-induced killer cells	Unknown, target *n* = 13	MRI scan for monitoring of tumour size and CIK cell homing	NCT01868490

Agents targeting BRAF mutations							
HM95573	RAF inhibitor	Solid tumours with RAS/RAF mutation, 2^nd^ line or more	I	HM95573	Recruiting, target *n* = 100	ORR	NCT03118817
Vemurafenib+HL-085	BRAF+MAPK inhibition	BRAF V600E mutant advanced solid tumours	I	Vemurafenib+HL-085	Recruiting, target *n* = 39	Incidence of AEs	NCT03781219
Vemurafenib	BRAF inhibitor	Tumours with BRAF mutation, 2^nd^ line or more	II Single-arm	Vemurafenib	Recruiting, target *n* = 500	ORR	NCT02304809
Encorafenib+MEK162	BRAF inhibitor+MEK inhibitor	BRAF V600E mutant advanced solid tumours	II Single-arm	Encorafenib+MEK162	Recruiting, target *n* = 179	DCR/ORR	NCT01543698
Dabrafenib+trametinib [ROAR]	BRAF inhibitor+MEK inhibitor	Rare tumours with BRAF V600E mutation, 2^nd^ line or more	II Open-label	Dabrafenib+trametinib	Completed, *n* = 206 (BTC, *n* = 35)	ORR	NCT02034110
Binimetinib+encorafenib [BEAVER]	BRAF inhibitor+MEK inhibitor	Advanced solid tumours with BRAF MT (non-V600E), 2^nd^ line or more	II Single-arm	Binimetinib+encorafenib	Not yet recruiting, target *n* = 26	ORR	NCT03839342

Agents targeting TRK aberrations							
Larotrectinib (BAY2757556) [NAVIGATE]	TRK inhibitor	Advanced solid tumours harbouring NTRK fusion, 2^nd^ line or more	II Single-arm	Larotrectinib	Recruiting, target *n* = 320	ORR	NCT02576431
Entrectinib (RXDX-101) [STARTRK-2]	NTRK1/2/3, ROS1, ALK	Advanced solid tumours that harbour NTRK1/2/3, ROS1, or ALK gene fusion	II Open-label	Entrectinib (RXDX-101)	Recruiting, target *n* = 300	ORR	NCT02568267

Agents targeting BAP1 aberrations							
Niraparib [UF-STO-ETI-001]	PARP inhibitor	Advanced solid malignancies with BAP1/DDR defects	II Nonrandomised, open-label	Niraparib	Recruiting, target *n* = 57	ORR	NCT03207347

Agents targeting HER2 aberrations							
DS-8201a [HERB]	HER2 inhibitor	HER2-positive biliary tract cancer	II Non-randomised	DS-8201a	Recruiting, target *n* = 32	ORR	JMA-IIA00423

GEM/CIS: gemcitabine+cisplatin; OS: overall survival; mFOLFIRINOX: modified FOLFIRINOX; FGFR: fibroblast growth factor receptor; TKI: tyrosine kinase inhibitor; ORR: overall response rate; PFS: progression-free survival; IDH: isocitrate dehydrogenase; MTD: maximum tolerated dose; AEs: adverse events; PARP: poly(ADP ribose) polymerase; PD1: programmed death 1; PDL1: programmed death ligand 1; CTLA4: cytotoxic T lymphocyte-associated protein 4; TMB-H: tumour mutation burden high; MAPK: mitogen-activated protein kinase; DCR: disease control rate; TRK: tropomyosin receptor kinase; ALK: anaplastic lymphoma kinase; BAP1: BRCA1-associated protein 1; DDR: DNA damage repair; PD: progressive disease.
